# Public mental health: a WPA priority and key opportunity to address implementation failure

**DOI:** 10.1007/s40211-025-00542-6

**Published:** 2025-09-01

**Authors:** Jonathan Campion, Afzal Javed

**Affiliations:** 1 Director for Public Mental Health and Consultant Psychiatrist, South London and Maudsley NHS Foundation Trust, London, UK; 2 Pakistan Psychiatric Research Centre, Fountain House, Lahore, Pakistan; 3 Honorary Professor of Public Mental Health, University of Cape Town, Cape Town, South Africa

**Keywords:** Public mental health, Prevention, Promotion, Implementation, Implementation failure, Öffentliche psychische Gesundheit, Prävention, Gesundheitsförderung, Implementierung, Mangelnde Behadlung

## Abstract

Mental health conditions (MHCs) account for a large proportion of global disease burden and result in broad impacts and associated economic costs. Despite the existence of effective public mental health (PMH) interventions, only a minority of individuals with MHCs receive treatment, far fewer receive interventions to prevent associated impacts, and there is negligible coverage of interventions to prevent MHCs or promote mental wellbeing and resilience. This implementation failure results in population-scale preventable suffering, broad impacts and associated economic costs, which are far greater in low- and middle-income countries. The gap also breaches the right to health and statutory legislation in some countries. The World Psychiatric Association has prioritised PMH and highlighted how a set of coordinated actions can improve PMH implementation, including by effectively making the PMH case, PMH practice, PMH training and improved population knowledge, settings-based and integrated approaches, digital technology, maximising existing resources, use of interventions with a large population impact, a rights approach and legislation, and implementation-focused research. Improved implementation results in broad population impacts, achievement of policy objectives across sectors and sustainable reduction in the impact of MHCs and promotion of population wellbeing. The associated economic benefits make PMH a key part of sustainable economic development.

## Abstract (de)

Psychische Erkrankungen inklusive subklinischer Syndrome sind weltweit Ursache für einen großen Anteil krankheitsbezogener Belastungen, die mit negativen Folgen und relevanten Kosten einhergehen. Obwohl wirksame Public-Health-Interventionen verfügbar sind, erhält nur eine Minderheit psychisch Erkrankter eine entsprechende Behandlung oder Interventionen, um die negativen Folgen zu vermeiden. Maßnahmen zur Prävention, zur Verbesserung von Wohlbefinden oder Stärkung der Resilienz werden noch seltener angeboten. Dieser Mangel an Umsetzung verursacht vermeidbares Leid, negative Krankheitsfolgen sowie beträchtliche Kosten, die in Ländern mit mittlerem oder geringem Einkommen höher sind als in Ländern mit hohem Einkommen. Das stellt auch eine Verletzung des Rechts auf Gesundheit dar. Der Weltverband für Psychiatrie (WPA) hat Public Mental Health zur Priorität erklärt und eine Reihe von koordinierten Schritten beschrieben, welche die Implementierung von Public-Mental-Health-Maßnahmen verbessern können, wie beispielsweise Ausbildung in Public Mental Health, Verbesserung des Wissens in der Bevölkerung, digitale Technologien, Vermehrung von Ressourcen, Interventionen mit großen populationsbezogenen Effekten, Anpassung der Gesetze und Forschung zur Implementierung. Eine verbesserte Umsetzung hat Wirkung in breiten Teilen der Bevölkerung und fördert eine nachhaltige Reduktion der negativen Folgen psychischer Erkrankungen sowie das Wohlbefinden in der Bevölkerung. Die damit verbundenen ökonomische Vorteile machen Public Mental Health zu einem wesentlichen Baustein einer nachhaltigen wirtschaftlichen Entwicklung.

## Introduction

This paper outlines the population impact of mental health conditions (MHCs) and wellbeing; it describes risk and protective factors, higher-risk groups, public mental health (PMH) interventions, and the implementation gap. It then highlights how a PMH approach can address PMH implementation failure, which results in broad impacts across sectors and associated economic benefits.

## Defining public mental health

Effective PMH interventions exist to treat mental health conditions (MHCs), prevent associated impacts, prevent MHCs from arising, and promote mental wellbeing and resilience. The World Psychiatric Association’s (WPA) Public Mental Health Working Group defined PMH practice as a population-based approach to mental health in order to improve the coverage, outcomes and coordination of PMH interventions, which, in turn, supports efficient, equitable, and sustainable reduction of MHCs and the promotion of mental wellbeing of populations [1].

**Table 1:** Primary, secondary and tertiary levels of mental health condition prevention, mental wellbeing promotion and resilience promotion [1]

Type of public mental health intervention	Levels of public mental health intervention
Prevention of mental health conditions	Primary level: interventions that prevent MHCs from arising
	Secondary level: early intervention for MHCs and associated impacts to minimise their effects
	Tertiary level: intervention for people with established MHCs to prevent relapse and the associated impacts in order to minimise disability
Promotion of mental wellbeing	Primary level: promotion of protective factors for mental wellbeing
	Secondary level: early promotion in people with recent deterioration in mental wellbeing
	Tertiary level: promotion in people with longstanding poor mental wellbeing
Promotion of resilience	Primary level: promotion of resilience
	Secondary level: early promotion of resilience in people with recent adversity
	Tertiary level: promotion of resilience in people with previous or longstanding adversity

## Mental health conditions, wellbeing and resilience

Types of mental health condition (MHCs) include affective disorders, anxiety disorders, post-traumatic stress disorder, eating disorders, psychosis, personality disorders, substance-use disorders, dementia and neurodevelopmental disorders. Types of mental wellbeing include affective (present satisfaction, pleasure and mood), evaluative (long-term life satisfaction and fulfilment in relation to specific aspects such as relationships, employment and community) and eudaimonic (meaning, purpose, value of one’s life) wellbeing [2]. Mental health conditions and wellbeing can be viewed as two related yet distinct continua, since MHCs do not preclude mental wellbeing.

Resilience involves the capacity to manage and adapt to different types of adversity.

## Impact of mental health conditions and wellbeing

Mental health conditions account for a large proportion of disease burden, which has been consistently underestimated [3]. This large proportion of disease burden occurs due to the combination of a high prevalence of MHCs; the early onset of MHCs in the life course; and the broad impacts across different sectors including health, education, employment, criminal justice and others which have large associated economic costs [1, 2]. These impacts are compounded by a large proportion of those with one MHC having another comorbid MHC as well as comorbid health risk behaviours and physical illness. A further large proportion of the population experiences subthreshold MHCs, where symptoms of MHCs do not reach the diagnostic threshold but result in considerable impact and an increased risk of developing MHCs.

Mental wellbeing also results in a broad range of impacts across health, including recovery from MHCs, education, employment and social interaction [2].

## Risk factors, protective factors and higher-risk groups

Particular factors are associated with increased risk of MHCs and poor wellbeing. Given that a large proportion of MHCs arise before adulthood, such factors are particularly important during childhood and adolescence. A population approach takes account of both the size of the impact and the proportion affected by each factor. During childhood, adversity has a large impact on the risk of MHCs [1, 4, 5]. Different MHCs are more robustly associated with particular risk factors [4]. The proportion of MHCs that can be attributed to a particular factor is referred to as the population-attributable fraction and informs how addressing several modifiable risk factors can reduce the incidence of different MHCs [5].

Certain risk factors have overarching impacts on other risk factors and include socioeconomic inequalities, conflict, environmental (pandemics, disasters, pollution, climate change) and commercial determinants (tobacco, alcohol, processed food, fossil fuels) [1, 6].

Particular protective factors are associated with better mental wellbeing [2].

Certain groups are at an increased risk of MHCs and poor mental wellbeing in part due to the clustering of risk factors in such groups [1, 6]. Examples include carers, particular ethnic groups, the homeless, people with a learning disability, lesbian gay bisexual queer transgender people (LBBTQ+), offenders, people subject to conflict, refugees and asylum seekers, and the unemployed.

## Public mental health interventions

Public mental health interventions can be divided into treatment of MHCs, prevention of associated impacts, prevention of MHCs, and promotion of mental wellbeing and resilience. Interventions may also be considered as primary, secondary, and tertiary levels of prevention and promotion (Table 1) or across the life course [1, 2]. People from higher-risk groups require more targeted PMH intervention to prevent widening of inequalities.

Many effective PMH interventions include cost–benefit evaluation, which demonstrates economics benefits even in the short term [2, 6].

Public mental health interventions are provided by different sectors including health, public health, social care, education, workplace, housing, criminal justice, the voluntary sector and others. This highlights the importance of coordinated approaches.

## Public mental health implementation gap

Despite the existence of evidence-based interventions, only a minority of people with MHCs receive any treatment [1, 7], and far fewer receive effective treatment [8]. There is even less coverage of interventions to prevent the associated impacts of MHCs, and negligible coverage of interventions to prevent MHCs or promote mental wellbeing and resilience. Coverage of PMH interventions is far less in low- and middle-income countries compared to high-income countries.

The PMH implementation gap breaches the right to health and results in population-scale preventable suffering, broad impacts and associated economic costs [1]. It is important to identify reasons for the scale of implementation failure which include insufficient PMH knowledge and training, insufficient mental health policy and transparency about associated implementation, insufficient resources, insufficient political will, the political nature of some PMH activities, and insufficient appreciation of cultural differences. Specific causes of the treatment gap include insufficient recognition of MHCs by the population and health workforce, insufficient training and delivery of evidence-based treatment, stigma, and insufficient understanding of the size of the treatment gap and ways to address it.

## Actions required to address public mental health implementation failure

Public mental health was prioritised in the WPA’s 2020–2023 Action Plan [9] as an important way to address implementation failure and remains prominent in WPA’s 2023–2026 Action Plan [10]. The following actions can address PMH implementation failure [1, 2] which are described in more detail in the next section:

1. Making the public mental health case
2. Public mental health practice
3. Public mental health training and improved population knowledge
4. Settings-based and integrated approaches
5. Digital technology
6. Maximising existing resources
7. Interventions with a large population impact
8. Rights approach and legislation
9. Implementation-focused research

### 1. Making the public mental health case

- Assessment of the PMH implementation gap is an important way to highlight the level of unmet need including for particular MHCs, stages of the life course and higher-risk groups. Public mental health needs assessment can occur at the following three levels:
  *Treatment of MHCs and prevention of associated impacts*: estimation of the prevalence and numbers affected by MHCs and associated impacts, followed by assessment of the proportion receiving interventions for treatment of MHCs and prevention of associated impacts by different providers.
  *Prevention of MHCs*: estimation of the prevalence and numbers affected by different risk factors for MHCs, followed by assessment of the proportion receiving interventions to address such factors by different providers.
  *Promotion of mental wellbeing and resilience*: estimation of the prevalence and numbers affected by different protective factors, followed by assessment of the proportion receiving interventions to address such factors by different providers.
  Public mental health needs assessment should also include the quality of implementation, expenditure on different PMH interventions by different sectors and the estimated economic benefits from improved intervention coverage. While assessments can be done at local, regional and national levels, national assessment is particularly important, since the size of the implementation gap at local and regional levels is closely relacted to the national implementation gap [1].
- *Coordinated advocacy and leadership*: coordinated advocacy and leadership is important in making the case given the impacts of MHCs and wellbeing across different sectors and since different types of PMH intervention are provided by various sectors. Coordinated advocacy with people with experience of MHCs is particularly important. Public mental health was a focus of the World Psychiatric Association’s 2020–23 Action Plan [9], which supported the development of a PMH Section by the European Psychiatric Association and a PMH Working Group by the World Federation of Public Health Associations. The WPA’s Public Mental Health Working Group and subsequent Special Interest Group has engaged with different sectors to support coordinated actions [11].
- *Public mental health economic case for investment*: advocacy and making the case can also be supported by estimation of the cost of implementation failure and economic benefits from scaled-up implementation [1, 12].

### 2. Public mental health practice

Public mental health practice involves the following three steps [1]:

- Use of the PMH needs assessment to inform:
  Choice of effective and implementable PMH interventions by different sectors.
  Coordinated policy and strategy across different sectors.
  Scaleup of implementation of effective PMH interventions.
  Transparent decisions about acceptable annual coverage of different PMH interventions by a range of stakeholders including people with lived experience and their carers. Such decisions should take account of the population-scale impact and cost of the PMH implementation gap, impacts and associated economic benefits of improved coverage, statutory legislation such as not to discriminate against people with MHCs, and the right to health.
  Resource and workforce required to implement agreed level of provision over specific timeframes.
  Communication with different sectors and wider population.
- Implementation of strategy and associated PMH interventions at agreed population coverage.
- Annual monitoring of coverage and outcomes of implementation, including for higher-risk groups.

### 3. Public mental health training and improved population knowledge

Insufficient PMH knowledge and training contributes to PMH implementation failure [1]. Key elements of PMH training include the impacts of MHCs and poor wellbeing; the prevalence of MHCs and poor wellbeing; risk and protective factors; higher-risk groups; effective PMH interventions provided by different sectors and their associated economic impacts; size of the PMH implementation gap and causes, including for higher-risk groups; and actions required to address PMH implementation failure [1, 6].

Public mental health training should be delivered to the workforce and leaders of different sectors, including treatment services, public health, education, employment, criminal justice, the voluntary sector, policymakers and people with lived experience of MHCs. More specific training for particular groups includes supporting the skills required for scaleup of delivery of PMH interventions by various sectors as well as development of PMH-informed policy by policymakers from different sectors. Public mental health training is also a recommended part of postgraduate psychiatry training [13].

Improved population knowledge about PMH supports early recognition and treatment of MHCs, reduced stigma, prevention of MHCs and promotion of mental wellbeing and resilience with more targeted approaches required for higher risk groups. Clear messaging including through digital and social media links to the public health agenda.

### 4. Settings-based and integrated approaches

Settings-based approaches offer opportunities to improve the coverage of different types of PMH interventions for particular sections of the population, e.g. antenatal settings, preschools and schools, workplaces and prisons. Integrated approaches both within and between sectors also supports coordinated delivery and improved coverage of PMH interventions.

### 5. Digital technology

This can support improved coverage of PMH interventions, PMH training [6] and interventions to promote PMH literacy.

### 6. Maximising existing resources

This can occur through self-help and use of digital interventions. Opportunities to maximise treatment resources include collaborative care, task sharing, improving the quality of treatment, non-specialist providers and peer support.

### 7. Use of interventions with a large population impact

Examples of interventions with potentially large population impacts on PMH include addressing [1, 2]

- Socioeconomic inequalities and poverty that underlie other risk factors and are also impacts of MHCs.
- Commercial determinants of health such as the price of alcohol, tobacco and unhealthy food [6, 14].
- Child adversity responsible for a large proportion of MHCs [5]; parenting programmes prevent both child adversity and child MHCs, are also first-line interventions for some child MHCs, and promote child behaviour, parental practice and mental health.
- Child and adolescent MHC treatment and prevention, given that the majority of lifetime MHCs arise before adulthood.
- Parental MHC treatment and prevention which can prevent childhood MHCs.
- Smoking cessation, which improves physical health/life expectancy and depression/anxiety [15].
- Physical inactivity [5].

### 8. Rights approach and legislation

The United Nations (UN) right to mental health is an important mechanism to advocate for appropriate coverage of PMH interventions and supports action to address the PMH implementation gap. Similarly, the UN Sustainable Development Goal of universal health coverage applies to mental health. Legislation can also support PMH implementation, including to prevent discrimination of people with MHCs, protect children and families from adversity, and promote workplace safety.

### 9. Implementation-focused research

Effective PMH interventions exist but are inadequately implemented. Research is required to support effective implementation in order to address population need, including for higher-risk groups in both high-income and low-and-middle-income countries.

## Conclusion

Mental health conditions result in broad impacts and associated economic costs. Despite the existence of effective PMH interventions, only a minority of individuals with MHCs receive treatment, far fewer receive interventions to prevent associated impacts, and there is negligible coverage of interventions to prevent MHCs, or promote mental wellbeing and resilience. This implementation failure breaches the right to health and results in population-scale preventable suffering, broad impacts and associated economic costs. A set of actions that improves implementation of PMH interventions results in broad population-scale impacts across sectors, achievement of a range of policy objectives across sectors, sustainable reduction in the impact of MHCs and promotion of population health. The associated economic benefits also make PMH a key part of sustainable economic development. The prioritisation of PMH by the World Psychiatric Association has supported a PMH focus in other organisations.
